# The challenge: streamlining HIV treatment and care while improving outcomes

**DOI:** 10.7448/IAS.17.4.19493

**Published:** 2014-11-02

**Authors:** Nathan Clumeck

**Affiliations:** Division of Infectious Diseases, Saint Pierre University Hospital, Brussels, Belgium

## Abstract

ART coverage among HIV-positive population varies, depending on the countries, between 10% (e.g. Indonesia) and 65% (Botswana). Death rates and new HIV infections have been linked to ART coverage. Therefore, streamlining tasks and roles to expand treatment and care and to provide quality and equitable health is an ongoing concern globally. One concept that has been applied to improve the delivery of HIV services is that of task shifting. Defined as the systematic delegation of tasks from doctors to cadre with less training such as nurses or lay workers, task shifting has been used as an effective strategy to address the current healthcare worker shortage in many African countries. A body of literature supports the use of task shifting as a successful approach in delivering healthcare services including HIV testing, counselling and ART treatment. In addition, in a time of economic burden and scare resources, task shifting may also help to relieve the situation. This concern is highlighted in recommendation of WHO to strengthen and expand human capacity among healthcare workers. The major issues that are raised are: How can task shifting be implemented in a way that is sustainable? How can clinical care services be organized to maximize the potential of the task-shifting approach while ensuring safety, efficiency and effectiveness? What preconditions must be met, what are the country-specific factors that will guide decision-making in the implementation of task shifting? In addition, task shifting should be implemented alongside other efforts to increase the numbers of skilled health workers. Quality assurances mechanisms should provide the necessary checks balances to protect both service users and health workers.

